# Genomic Sequence of a Swine Pasivirus Type 1 Strain Identified in U.S. Swine

**DOI:** 10.1128/genomeA.01569-17

**Published:** 2018-02-08

**Authors:** Baoqing Guo, Hanjun Kim, Ying Zheng, Huigang Shen, Roman M. Pogranichniy, Kent J. Schwartz, Ganwu Li, Kyoung-Jin Yoon

**Affiliations:** a Department of Veterinary Diagnostic and Production Animal Medicine, College of Veterinary Medicine, Iowa State University, Ames, Iowa, USA; b Department of Comparative Pathobiology, College of Veterinary Medicine, Purdue University, West Lafayette, Indiana, USA

## Abstract

We report for the first time in the United States the identification of a swine pasivirus (SPaV) strain with a genomic sequence identity of less than 80% to other SPaVs reported in Europe and China, using a next-generation sequencing (NGS) technique in sow tissues collected from an animal study conducted in 2001, suggesting virus circulation in domestic swine.

## GENOME ANNOUNCEMENT

Swine pasivirus (SPaV) is a relatively new member of the family *Picornaviridae*. The *Pasivirus* genus was recently formed in group 4 of this family, with swine pasivirus type 1 (SPaV1) as the proposed type species, which is now *Pasivirus A* (1); SPaV1 was first identified in fecal samples of healthy piglets in France in 2012 (2). Subsequently, similar viruses were reportedly identified in China, Hungary, Germany, and Romania (3–5). Here, we report the identification of SPaV1 in U.S. swine.

In an effort to identify unknown viral agents in pig tissues derived from a previous animal study conducted in 2001 concerning porcine reproductive and neurological syndrome (6), next-generation sequencing (NGS) was attempted. Various tissues (lung, lymph node, spleen, and tonsil) from sows were processed to prepare tissue homogenates, which were then clarified by low-speed centrifugation and concentrated by ultracentrifugation for nucleic acid extraction for RNA viruses. The nucleic acid extracts were used to make NGS libraries according to the manufacturer’s instructions using the TruSeq stranded total RNA library prep kit (Illumina, Inc., San Diego, CA). Sequencing was performed on a MiSeq system (Illumina, Inc.), as previously described (7). Sequences obtained from the MiSeq run were analyzed using Kraken (8), and viral genome sequences of interest were *de novo* assembled by a pipeline developed in-house, as described previously (7).

Unexpectedly, an almost full-length pasivirus genome of 6,695 nucleotides was obtained from a sow tissue pool. The virus strain was designated as SPaV1/US/17-50816IA60467-1/2001 (here, US SPaV1). Analysis of the US SPaV1 sequence demonstrated that the virus has a genomic organization similar to that of prototype SPaV1 (GenBank accession no. JQ316470), in which 6,412 nucleotides of the genome encode a polyprotein flanked by 5ʹ- and 3ʹ-noncoding sequences. A pairwise comparison of the polyprotein-coding sequences between US SPaV1 and other swine pasiviruses whose sequences were available in GenBank (https://www.ncbi.nlm.nih.gov/genbank/) revealed a nucleotide identity from 76.6% to 81.0%, while the deduced amino acid sequences had 82.1% to 89.7% identity among them. VP1 shares 73.4 to 79.8% nucleotide (nt) identity and 76.4 to 90.9% amino acid (aa) identity among them. In a sequence comparison of the 5ʹ-untranslated region (UTR), a putative internal ribosome entry site (IRES) located there showed 94.7% to 98.2% identity between the 5ʹ-UTR of US SPaV1 and other pasivirus sequences.

The identification of SPaV1 in U.S. swine shows a broader global distribution of the virus. As the animal study (6) was actually conducted in 2001, SPaV1 may have circulated in swine populations much longer than previously believed. More research will be needed to elucidate its evolution, diversity, and health significance.

### Accession number(s).

The complete polyprotein-coding sequence of U.S. swine pasivirus type 1 (SPaV1/US/17-50816IA60467-1/2001) has been submitted to GenBank under the accession number MG674090.
